# Association among smoking, depression, and anxiety: findings from a representative sample of Korean adolescents

**DOI:** 10.7717/peerj.1288

**Published:** 2015-10-01

**Authors:** Haewon Byeon

**Affiliations:** Department of Speech Language Pathology & Audiology, Nambu University, Gwangju, Republic of Korea

**Keywords:** Depression, Cigarette smoking, Anxiety, Mental health, Adolescents

## Abstract

This study investigated the relationship between smoking and depression and anxiety using data from a nationwide survey representing Korean adolescents. Subjects were 6,489 adolescents in middle and high school (age 13–18) who had participated in the 2011 Korean Study of Promotion Policies on Children and Adolescents—Mental Health (KSPCAM). Daily smoking number of times for current smokers was classified as 1–2 times, 2–4 times and over 5 times. The odds ratio for the statistical test was presented using hierarchical logistic regression. When adjusted for covariates (gender, age, household economy, type of residing city, type of school, school record, satisfaction with school life, subjective health status, satisfaction with relationship with parents, and drinking experience), smokers more significantly likely to have depression (OR = 1.27, 95% CI [1.02–1.57]), and anxiety (OR = 1.49, 95% CI [1.14–1.96]) than non-smokers (*p* < 0.05). In addition, adolescents who smoke more than 5 cigarettes a day were 1.5 times more likely to have depression (OR = 1.48, 95% CI [1.13–1.92]) and anxiety (OR = 1.49, 95% CI [1.07–2.08]) than those who do not smoke. Smoking in adolescence was found to be significantly related with depression and anxiety. To promote the mental health of adolescents, effective smoking cessation programs are required.

## Introduction

Although numerous studies have consistently reported on the relationship between smoking and diseases over the last decade, the smoking rate in Korean adolescents (> age 15) has decreased less than 2% from 16.6% in 2005 to 14% in 2013. As of 2013, the smoking rate of Korean adolescents was approximately twice that of Canadian adolescents (OECD, 2013). In addition, the daily smoking rate of high school students (i.e., the percentage of students who have smoked every day for the last 30 days) was 7.4% in Korea as of 2013, 1.8 times higher than that of 2005 (Korea Centers for Disease Control and Prevention, 2009). In sum, one out of every ten Korean adolescents is a current smoker, and half of these smokers smoke every day.

Smoking is well known as a health risk behavior. A cigarette contains more than 69 kinds of carcinogens, such as benzopyrene, and more than 4,000 kinds of chemicals. Excessive smoking causes not only respiratory diseases (Zhang, 2014) but also lung cancer (Jee et al., 2004). According to a report by the WHO, smoking is the number-one cause of death, and as of 2012, one out of every ten deaths was related to smoking (World Health Organization, 2012). In addition, recent studies have reported that smoking has negative effect on mental health (Martinez-Hernaez & Abbas, 2015; Mayfield Arnold et al., 2014; Murphy et al., 2003). Therefore, smoking policy is required on a national level to promote public health.

As adolescence is a transitional period between childhood and adulthood involving both physical and psychological growth, early management of health risk behaviors, such as smoking, is important. Adolescent smokers have a high probability of continuing into adulthood (Mcgue & Lacono, 2005), and the younger a person starts smoking, the greater the nicotine dependency, which makes quitting more difficult (Korea Centers for Disease Control and Prevention, 2013). Moreover, it has been shown that the younger a smoker starts smoking, the greater the effect of smoking on the reduction of lifespan. It has been reported that smoking that starts at the age of 25 reduces lifespan by four years, while smoking that starts at the age of 15 reduces lifespan by eight years (Fielding, Husten & Eriksen, 2008). For these reasons, many countries have defined smoking as a major health risk and are attempting to manage it at the state level. For example, the U.S. Centers for Disease Control and Prevention (CDC) established the Youth Risk Behavior Surveillance System and set reduction of the smoking rate as one of the seven major goals for the promotion of adolescent health (Kann, Kolbe & Collins, 1993). The Korean Government also established the National Health Promotion Plan 2020 in 2011 and implemented an anti-smoking policy with the goal of reducing the male smoking rate from 46.9% in 2009 to 29% by 2020 (Park, 2010). Nevertheless, in Korea, not only are adolescents starting smoking younger but the smoking rate is increasing with age as well (Korea Centers for Disease Control and Prevention, 2009).

Meanwhile, numerous epidemiological studies have reported that smoking affects physical health as well as mental health (Chation et al., 2009; Gilpin, Lee & Pierce, 2004; Lam et al., 2005; Park et al., 2010; Patton et al., 1998; Richardson et al., 2012; Wu & Anthony, 1999). According to such studies, smokers have a 1.9–2.8 times greater risk of depression than non-smokers (Brown et al., 1996; Richardson et al., 2012). On the other hand, other studies have reported that smoking is not related with depression or anxiety (Takemura et al., 1999; Williams & Adams-Campbell, 2000). In Breslau & Johnson’s study (2000), smokers’ nicotine dependency was not found to be significantly related with depression (Breslau & Johnson, 2000). However, few of these studies considered school and family environment factors that may affect adolescents’ mental health. In particular, unlike those of adults, the emotions of adolescents are affected by health factors as well as school environment factors, such as school record and satisfaction with school life.

If adolescents’ mental health problems are neglected, they are highly likely to continue into adulthood, which is associated with an enormous social cost. Therefore, the mental health problems of adolescents should be recognized as a social problem rather than an individual one, and the analysis of the relationship between smoking and depression and anxiety is important when investigating risk factors for adolescent mental health problems.

Therefore, this study investigated the relationship between smoking and depression and anxiety using data from a nationwide survey representing Korean adolescents.

## Methods

### Study population

The source of the data was the 2011 Korean Study of Promotion Policies on Children and Adolescents—Mental Health (KSPCAM), a nationwide survey on non-institutionalized adolescents in local communities of South Korea conducted by the National Youth Policy Institute under the umbrella of the Prime Minister’s Office. The survey was approved by the IRB of the National Youth Policy Institute (No. 2011-KSPCAM-1015). The sampling design and administration of the KSPCAM are described in detail elsewhere (National Youth Policy Institute, 2013). In brief, the KSPCAM was conducted for the purpose of investigating the mental health status of Korean adolescents and preparing a policy improvement plan to promote adolescents’ mental health. The population included primary, middle, and high school students from 16 cities and provinces across the country, and stratified multistage cluster sampling was employed based on the 2011 Statistical Yearbook of Education. The 2011 survey was conducted by researchers who visited 300 sample schools in person to conduct the survey using paper-and-pencil interviews. Areas of the survey were classified into individuals, families, local communities, and schools, and questions addressed mental health, satisfaction with life, and school life. Before the survey, basic information (e.g., school name, contact address and phone number, suitability of target schools for survey such as closing of schools, redundancy of target schools) was carefully checked, and an official letter requesting cooperation and information sheets were sent to deputy principals of the target schools in advance to induce cooperation. In addition, in order to minimize non-sampling error and enhance the accuracy of the survey, three education and training sessions were conducted with researchers and coding experts, and two reviews of the surveyed data were conducted by coding experts.

The subjects of this study were 6,492 adolescents in middle and high school (age 13–18) who had participated in the 2011 KSPCAM. Finally, a total of 6,489 adolescents (3,352 males, 3,137 females) were analyzed after the exclusion of three adolescents who could not finish the questionnaires.

### Measurement

#### Smoking

For smoking, life-time smoking experience and average number of cigarettes smoked per day were surveyed. Life-time smoking experience was classified into smokers (a person who at the time of the survey, smokes any tobacco product either daily or occasionally) and non-smokers (a lifetime nonsmoker) by referring to World Health Organization (1998) standards. Average number of cigarettes smoked per day was classified as 1–2 cigarettes, 2–4 cigarettes and over 5 cigarettes.

#### Depression

Depression was measured using Beck‘s Depression Inventory (BDI) (Beck, 1991). BDI is a self-administered questionnaire test with 21 questions on emotional, cognitive, motivational and physiological symptoms and it is composed of 4-point scale from ‘Not at all (0 point)’ through ‘Very much so (3 points)’. Total points were 63 and cut-off point for depression was set over 21 points. Cronbach’s alpha which shows internal validity was 0.91.

#### Anxiety

For anxiety, Beck Anxiety Inventory (BAI) (Hahn et al., 1986) was used which is also a self-administered questionnaire survey. BAI is composed of 21 questions and 4-point scale from ‘Not at all (0 point)’ through ‘Very much so (3 points)’. Total points were 63 and cut-off point for anxiety was set over 22 points. Cronbach’s alpha was 0.92.

#### Confounding factors

Confounders included gender, age, household economy (high, medium, low), type of residing city (metropolitan, medium and small city, rural area), type of school (middle, high, vocational high school), school record (high, medium, low), satisfaction with school life (satisfied, average, dissatisfied), subjective health status (good, normal, pool), satisfaction with relationship with parents (satisfied, average, dissatisfied) and drinking experience (yes, no). Residing city was classified into city and rural area based on administrative classification and city was classified into ‘metropolitan’ when it is with population over 1 million and ‘medium and small’ when with population of from 100,000 through 1 million. Household economy was classified into 3 brackets by defining them with variable of household income.

### Statistical analysis

Chi-square tests were used to compare the smoking rates by demographics, lifestyles, and health factors. For the relationship between smoking and depression, odds ratio and 95% confidence intervals were presented. Model 1 was adjusted with gender, age, household economy, residing city, school record and satisfaction with school while Model 2 was additionally adjusted with confounders including subjective health status, satisfaction with parents and drinking experience. All analyses were conducted using MINITAB version 13 (Minitab Inc., State College, Pennsylvania, USA).

## Results

### General characteristics of subjects related to life-time smoking

The general characteristics of subjects related to smoking are presented in Table 1. As the result of independent *t*-test, average age of smokers (16.0 years old) was higher than non-smokers (15.6 years old) (*p* < 0.05). As the result of Chi-square test, there were significant differences between the smokers and non-smokers in gender, household economy, residing city, school record, satisfaction with school life, satisfaction with relationship with parents, subjective health status, drinking experience, depression and anxiety (*p* < 0.05). Smoking rate was higher in males (28.5%), group with low household economic status (26.5%), rural areas (26.5%), vocational high schools (35.7%), adolescents with low school records (27.9%), adolescents who are dissatisfied with school life (26.4%), adolescents with poor subjective health (27.2%), adolescents dissatisfied with their relationship with parents (29.0%), adolescents with drinking experience (41.1%), adolescents with depression (27.8%) and adolescents with anxiety disorder (32.8%).

**Table 1:** Characteristics of subjects based on life-time smoking, *n* (%).

Variables	Non-smokers (*n* = 5,185)	Smokers (*n* = 1,304)	*p*
Age (mean ± SD)	15.6 ± 1.7	16.0 ± 1.6	<0.001
Sex			<0.001
Male	2,397 (71.5)	955 (28.5)	
Female	2,788 (88.9)	349 (11.1)	
Economics home (Tertile)			<0.001
High	643 (81.2)	149 (18.8)	
Median	3,363 (82.0)	737 (18.0)	
Low	1,162 (73.5)	418 (26.5)	
Type of residing city			<0.001
Metropolitan	2,169 (78.6)	591 (21.4)	
Medium and small city	2,510 (82.5)	531 (17.5)	
Rural area	506 (73.5)	182 (26.5)	
Type of school			<0.001
Middle school	2,388 (84.0)	454 (16.0)	
High school	1,932 (83.9)	370 (16.1)	
Vocational high school	865 (64.3)	480 (35.7)	
School record (Tertile)			<0.001
High	1,385 (86.3)	219 (13.7)	
Median	2,077 (83.2)	418 (16.8)	
Low	1,718 (72.1)	666 (27.9)	
Satisfaction with school life			<0.001
Satisfied	1,782 (83.2)	360 (16.8)	
Average	2,139 (81.2)	494 (18.8)	
Dissatisfied	1,255 (73.6)	450 (26.4)	
Self-reported health status			<0.001
Good	1,432 (82.9)	296 (17.1)	
Normal	2,827 (80.9)	667 (19.1)	
Pool	821 (72.8)	306 (27.2)	
Satisfaction with relationship with parents			<0.001
Satisfied	2,512 (84.4)	463 (15.6)	
Average	1,502 (80.7)	360 (19.3)	
Dissatisfied	1,090 (71.0)	446 (29.0)	
Drinking experience			<0.001
Yes	1,580 (58.9)	1,104 (41.1)	
No	3,601 (94.8)	199 (5.2)	
Depression			<0.001
Yes	879 (72.2)	338 (27.8)	
No	4,256 (81.8)	947 (18.2)	
Anxiety			<0.001
Yes	353 (67.2)	172 (32.8)	
No	4,755 (81.1)	1,106 (18.9)	

### The relationship between smoking and depression

The relationship between smoking and depression is presented in Table 2. As the result of hierarchical logistic regression analysis, smoking had significant relationship with depression in all models of this study (*p* < 0.05). When all confounders were adjusted in model 2, smokers were 1.3 times (OR = 1.27, 95% CI [1.02–1.57]) more significantly likely to have depression than non-smokers (*p* < 0.05). In addition, adolescents who smoke more than 5 cigarettes a day were 1.5 times (OR = 1.48, 95% CI [1.13–1.92]) more significantly likely to have depression (*p* < 0.05).

**Table 2:** Hierarchicallogistic regression analyses of the association between the smoking and depression: odds ratio (OR) and confidence interval (CI).

Smoking	Univariate model	Model 1	Model 2
Non smoker	1	1	1
Smoker	1.73 (1.50, 1.99)^*^	1.59 (1.34, 1.88)^*^	1.27 (1.02, 1.57)^*^
Cigarette per day			
1–2	1.33 (1.04, 1.71)^*^	1.22 (0.93, 1.62)	1.10 (0.81, 1.50)
3–4	1.06 (0.62, 1.83)	0.88 (0.48, 1.61)	0.92 (0.49, 1.75)
≥5	2.03 (1.71, 2.40)^*^	1.91 (1.57, 2.34)^*^	1.48 (1.13, 1.92)^*^

**Notes.**

* *p* < 0.05.

Model 1: Adjusted for sex, age, type of school, economics home, school record, and satisfaction with school life.

Model 2: Additionally adjusted for self-reported health status, satisfaction with relationship with parents, and drinking experience.

### The relationship between smoking and anxiety

The relationship between smoking and anxiety is presented in Table 3. In all models of the study, smoking had significant relationship with anxiety. In model 2, when all confounders were adjusted, smokers were 1.5 times (OR = 1.49, 95% CI [1.14–1.96]) more significantly likely to have anxiety than non-smokers (*p* < 0.05) and adolescents who smoke more than 5 cigarettes a day were 1.5 times (OR = 1.49, 95% CI [1.07–2.08]) more significantly likely to have anxiety (*p* < 0.05).

**Table 3:** Hierarchicallogistic regression analyses of the association between the smoking and anxiety: odds ratio (OR) and confidence interval (CI).

Smoking	Univariate model	Model 1	Model 2
Non smoking	1	1	1
Smoking	2.09 (1.73, 2.54)^*^	2.13 (1.71, 2.65)^*^	1.49 (1.14, 1.96)^*^
Cigarette per day			
1–2	1.96 (1.42, 2.69)^*^	1.95 (1.39, 2.74)^*^	1.62 (1.12, 2.36)^*^
3–4	1.12 (0.52, 2.45)	1.06 (0.47, 2.38)	1.03 (0.44, 2.39)
≥5	2.28 (1.82, 2.86)^*^	2.39 (1.85, 3.09)^*^	1.49 (1.07, 2.08)^*^

**Notes.**

* *p* < 0.05.

Model 1: Adjusted for sex, age, type of school, economics home, school record, and satisfaction with school life.

Model 2: Additionally adjusted for self-reported health status, satisfaction with relationship with parents, and drinking experience.

## Discussion

In the present epidemiological study, smoking was found to be significantly related with anxiety and depression in adolescence. Even after confounders were adjusted, smokers were 1.3 times more likely to have depression and 1.5 times more likely to have anxiety than lifetime non-smokers. As for the relationship between mental health and smoking, numerous longitudinal studies and meta-studies have verified that depression and stress are independent risk factors related with the initiation of smoking (Chation et al., 2009; Gilpin, Lee & Pierce, 2004; Lam et al., 2005; Martinez-Hernaez & Abbas, 2015). On the contrary, there have been large-scale epidemiological studies on adolescents that have reported that smoking may influence depression (Park et al., 2010; Patton et al., 1998; Wu & Anthony, 1999). According to a cross-sectional study by Richardson et al. (2012) conducted on 1,884 adolescents aged 12–15 who participated in the U.S. National Health and Nutrition Survey performed from 1999 through 2004, after the control of socio-demographic factors, smokers were 2.8 times more likely to suffer from depression than non-smokers. In addition, in a study on 1,709 adolescents aged 14–18, smokers were 1.9 times more likely to suffer from major depressions than non-smokers (Brown et al., 1996). Especially, the younger the smoker, the higher the risk of depression by smoking; in case of young adolescents aged 12 through 14, smokers were 4 times more likely to have depressive symptoms than non-smokers (Mayfield Arnold et al., 2014). This causal relationship between smoking and depression has been verified by longitudinal studies conducted on populations of various ethnicities. In a prospective cohort study that traced 2,032 Australian adolescents aged 14–15 over a three-year period, Patton et al. (1998) found that smokers had a 2 times greater risk of depression. In addition, in a study that traced 1,731 Chinese adolescents aged 13–14 for nine years, Wu & Anthony (1999) found that smokers had a 1.7 times greater risk of depression, and in a longitudinal study, Park et al. (2010) found that Korean adolescents who continued smoking into adulthood had significantly higher levels of depression than non-smoking adolescents. Lee et al. (1994) conducted an analysis of depression-related factors in university freshmen under the age of 20 by using a standardized depression test tool, the Center for Epidemiological Studies-Depression Scale (CES-D). The results showed that smokers were 1.5 times more likely to be at risk of pathological depression than non-smokers. Behavioral and physical symptoms—including withdrawal, tolerance, and cravings—are included in the diagnosis standards for tobacco use disorders prescribed as diseases by the DSM-5 (American Psychiatric Association, 2013). These consistent results of cross-sectional and longitudinal studies support the results of this study that smoking is significantly related with depression. Based on the results of this study, investigations into the realities of smoking adolescents’ mental health and their continuous management are required.

The effect of smoking on depression and anxiety can be explained by the neuro-biochemical response mechanism of nicotine and monoamine oxidase. First, smoking increases the amount of nicotine in the blood. Then, nicotine promotes corticosteroid, a hormone in the body, by stimulating the hypothalamic-pituitary-adrenal axis (HPA axis) and increases the manifestation of messenger RNA related to steroids in the cells of the cerebral amygdala, which is in charge of emotions and feelings (Haustein, Haffner & Woodcock, 2002). This series of actions is similar to the acute stress response (Kim & Kim, 2007). Second, smoking is also associated with the decline of monoamine oxidase. In a recent systematic exploratory study (Rendu et al., 2011), it was found that monoamine oxidase, which is responsible for the decomposition of amine in the human body, is less activated in smokers than in non-smokers. To sum up, sustained smoking affects adolescents’ depression and anxiety, as it increases the amount of nicotine in the blood and decreases the amount of monoamine oxidase, both of which are closely related to depression.

Meanwhile, this study found that daily average levels of smoking were associated with depression and anxiety among adolescents. Adolescents who smoked more than five cigarettes a day were 1.5 times more likely to suffer from depression and anxiety. Numerous epidemiological studies have reported that smoking is in a dose–response relationship with depression and anxiety. It has been reported that as the smoking period increases, the likelihood of depression symptoms increases (Martini, Wagner & Anthony, 2002) and that as the nicotine addiction increases in severity, the risk of depression also increases (Khaled et al., 2009). According to a study by Nelson & Wittchen (1998), nicotine addicts are over 4 times more likely to suffer from major depression disorders than non-smokers. In addition, smoking more than five cigarettes a day is significantly related with anxiety. In an epidemiological study on American adolescents, heavy cigarette smokers who smoked more than 20 cigarettes a day had a 6.7 times greater risk of agoraphobia, a 5.5 times greater risk of generalized anxiety disorder, and a 15.6 times greater risk of panic disorder than non-smokers (Johnson et al., 2000). It is difficult to directly compare these results with those of this study, since there is a lack of preceding studies on the relationship between daily average smoking amount and adolescent mental health. However, as the relationship between habitual smoking and depression and anxiety has been verified, extra attention must be paid to the mental health of smoking adolescents.

The limitations of this study are as follows: First, potential confounders that affect depression and anxiety may exist other than the ones included in this study. In particular, as this study did not address depression-related medical histories, future studies should include diseases related to depression as confounders and investigate their relationship with smoking. Second, even though this study verified the independent relationship between smoking and depression, it cannot be considered a causal relationship, as the result is based on a cross-sectional study over a specific period of time. In order to determine a causal relationship, longitudinal studies are required.

## Conclusion

Smoking in adolescence was found to be significantly related with depression and anxiety. Smoking is a preventable health-risk behavior. To promote the mental health of adolescents, effective smoking cessation programs are required.

## Supplemental Information

10.7717/peerj.1288/supp-1 Supplemental Information 1 Raw Data—2011 Korean Study of Promotion Policies on Children and Adolescents—Mental Health The source of the data was the 2011 Korean Study of Promotion Policies on Children and Adolescents—Mental Health (KSPCAM), a nationwide survey on non-institutionalized adolescents in local communities of South Korea conducted by the National Youth Policy Institute under the umbrella of the Prime Minister’s Office.

## Additional Information and Declarations

### Data Availability

The following information was supplied regarding data availability: 2011 Korean Study of Promotion Policies on Children and Adolescents—Mental Health: http://www.nypi.re.kr.
